# Self-assembling functional programmable protein array for studying protein–protein interactions in malaria parasites

**DOI:** 10.1186/s12936-018-2414-2

**Published:** 2018-07-17

**Authors:** Gabriela Arévalo-Pinzón, María González-González, Carlos Fernando Suárez, Hernando Curtidor, Javier Carabias-Sánchez, Antonio Muro, Joshua LaBaer, Manuel Alfonso Patarroyo, Manuel Fuentes

**Affiliations:** 1 Fundación Instituto de Inmunología de Colombia (FIDIC), Carrera 50 # 26-20, Bogotá, Colombia; 2 PhD Programme in Biomedical and Biological Sciences, Universidad del Rosario, Carrera 24 # 63C-69, Bogotá, Colombia; 3 Proteomics Unit, Cancer Research Centre (IBMCC/CSIC/USAL/IBSAL), 37007 Salamanca, Spain; 4 Department of Medicine and General Cytometry Service-Nucleus, Cancer Research Centre (IBMCC/CSIC/USAL/IBSAL), 37007 Salamanca, Spain; 5 Universidad de Ciencias Aplicadas y Ambientales (U.D.C.A.), Calle 222 # 55-37, Bogotá, Colombia; 6 School of Medicine and Health Sciences, Universidad del Rosario, Carrera 24 # 63C-69, Bogotá, Colombia; 7 Unidad de Investigación Enfermedades Infecciosas y Tropicales (e-INTRO), Instituto de Investigación Biomédica de Salamanca-Centro de Investigación de Enfermedades Tropicales de la Universidad de Salamanca (IBSAL-CIETUS), Facultad de Farmacia, Universidad de Salamanca, Campus Universitario Miguel de Unamuno s/n, 37007 Salamanca, Spain; 8 Virginia G. Piper Center for Personalized Diagnostics, Biodesign Institute, Arizona State University, Tempe, AZ USA

**Keywords:** *Plasmodium vivax*, Malaria, NAPPA array, IVTT protein expression, Protein–protein interaction

## Abstract

**Background:**

*Plasmodium vivax* is the most widespread malarial species, causing significant morbidity worldwide. Knowledge is limited regarding the molecular mechanism of invasion due to the lack of a continuous in vitro culture system for these species. Since protein–protein and host–cell interactions play an essential role in the microorganism’s invasion and replication, elucidating protein function during invasion is critical when developing more effective control methods. Nucleic acid programmable protein array (NAPPA) has thus become a suitable technology for studying protein–protein and host–protein interactions since producing proteins through the in vitro transcription/translation (IVTT) method overcomes most of the drawbacks encountered to date, such as heterologous protein production, stability and purification.

**Results:**

Twenty *P. vivax* proteins on merozoite surface or in secretory organelles were selected and successfully cloned using gateway technology. Most constructs were displayed in the array expressed in situ, using the IVTT method. The *Pv*12 protein was used as bait for evaluating array functionality and co-expressed with *P. vivax* cDNA display in the array. It was found that *Pv*12 interacted with *Pv*41 (as previously described), as well as *Pv*MSP1_42kDa_, *Pv*RBP1a, *Pv*MSP8 and *Pv*RAP1.

**Conclusions:**

NAPPA is a high-performance technique enabling co-expression of bait and query in situ, thereby enabling interactions to be analysed rapidly and reproducibly. It offers a fresh alternative for studying protein–protein and ligand–receptor interactions regarding a parasite which is difficult to cultivate (i.e. *P. vivax*).

**Electronic supplementary material:**

The online version of this article (10.1186/s12936-018-2414-2) contains supplementary material, which is available to authorized users.

## Background

Malaria is one of the most important tropical diseases transmitted by vectors worldwide; *Plasmodium vivax* represents one of the most widely distributed species (affecting ~ 13.8 million people worldwide per year). Despite this, the apparently slow progress of infection and low parasitaemia levels in humans compared to those reported in *Plasmodium falciparum* have erroneously led to *P. vivax* infection being classified as benign. Added to this, the experimental challenges involved in culturing this parasite greatly hinder accumulating the biological, cellular and molecular knowledge necessary for developing effective control methods against *P. vivax*. Although *P. vivax* invasion is thought to be similar to that of *P. falciparum*, some biological differences condition disease severity and hamper its biological study [1, 2].

*Plasmodium* parasites have a diverse ligand repertoire [3] and adapt to differing conditions [4, 5]; however, the scenario is much more complex. Evidence has grown regarding macromolecular complex formation between ligands [6–8] and host surface molecule multimeric assemblies which could favour and increase the strength of any receptor–ligand interaction [6–13]. However, little is known about macromolecular complexes and host–pathogen interactions concerning *P. vivax* [14]. Some proteins’ functions have been speculated about to date on the basis of their counterparts in other species [15], mainly due to this species preferential invasion of young RBC, meaning that no continuous in vitro culture is available for real-time evaluation of protein interactions during invasion [2]. Moreover, a preliminary characterization of such multimeric complexes has also been hampered by the technical challenges when expressing recombinant proteins in an active, soluble and immunogenic form in cell-based expression systems (CBES), particularly for the extracellular ones which might participate during merozoite invasion of target cells. Several factors negatively affecting obtaining *Plasmodium* proteins in CBES have been described, such as the existence of long stretches of repeated amino acid sequences, the high isoelectric point, and the presence of signal peptide, GPI anchor or transmembrane regions [16, 17]. In addition, the high AT-content results in low codon usage compatibility in heterologous expression systems, such as *Escherichia coli* [18]. Other factors like the presence of disulfide bridges encompassing important structural domains represent a challenge when obtaining properly folded proteins. In contrast to CBES, cell-free expression systems (CFES) based on eukaryotic or prokaryotic cell extracts (i.e. *E. coli*, wheat germ extract (WGE), rabbit reticulocyte lysate (RRL), HeLa, etc.) [19–22] offer a viable alternative for expressing soluble *Plasmodium* proteins displaying the proper conformation [23–26]. Moreover, the CFES substantial time-savings (2 h vs. 24–48 h for protein expression), have the ability to adapt to high-throughput formats, increased tolerance to additives and less sensitivity to toxic or proteolytic proteins when compared to CBES [27]. In previous study, WGE was used for expressing 89 soluble *P. vivax* proteins and analysing the immunoproteome, four potential antigens (including Pv24) were identified [28]. Other study, comparing the expression of five *P. vivax* vaccine candidate antigens between extracts from prokaryotic (*E. coli*) and WGE cells, has shown that despite both systems allow producing soluble proteins that are easily detectable, proteins produced in the eukaryotic system were recognized by a greater number of sera from *P. vivax*-infected patients than identical proteins produced in *E. coli* extracts [29]. Together, these data showed that CFES, particularly the eukaryotic ones, represent a good choice for studying *P. vivax* proteins.

Interestingly, CFES have been successfully coupled to both solid and suspension arrays, mainly being used for to identify novel blood-stage malaria vaccine candidates through antibody reactivity from adults who lived in a malaria endemic area [30–32]. In this approach, CFES-expressed polypeptides are then printed on the array. This methodology, however, has some drawbacks such as the requirement of maintaining cold-chain after protein expression to avoid losing protein function and that printing thousands of recombinant proteins on a chip is a laborious task, reduces reproducibility and increases costs.

Several arrays that allow protein expression in situ have been developed during the last few years to overcome the above-mentioned problems, such as DNA-arrays to protein-arrays (DAPA), Protein in situ arrays (PISA) and Nucleic Acids Programmable Protein Arrays (NAPPA). NAPPA produces protein microarrays using CFPS (mainly RRL) to transcribe and translate cDNA-encoded bait proteins directly onto glass slides [29, 33]. De novo synthesized proteins are directly captured by anti-tag antibodies co-spotted with specific cDNA. A target/bait in vitro transcription/translation (IVTT) expressed protein is thus captured onto the surface ready for functional assays; this approach has been successfully used in applications such as vaccine development and evaluating autoimmune responses and protein–protein interactions [34–36]. A query protein is simultaneously IVTT co-expressed with target/bait proteins in protein interaction assays; however, this approach requires a different C-terminal tag. Such approach has been successfully used in several applications, i.e. determining *Legionella pneumophila* effector (SidM and LidA) interaction network with 10,000 unique human proteins [36] and evaluating anti-serum profiles and protein interactions in a cDNA expression library from *Ornithodoros moubata* salivary glands [37].

The difficulty of maintaining a continuous *P. vivax* culture hampers evaluating protein–protein interactions when using functional approaches such as knockouts (total, conditional with or without complementation) [4, 38], knockdowns or inhibition assays [39]. Alternatives are needed and NAPPA technology thus stands out as a very good choice for studying *P. vivax* host–pathogen and protein–protein interactions. The present study involved designing and developing a NAPPA array constructed by non-contact printing of 20 *P. vivax* key genes encoding proteins which might be involved in Mrz invasion of reticulocytes.

Each gene was sub-cloned in a vector compatible with the IVTT expression system encoding a C-terminal tag. Such array has been optimized and fully-characterized to study in situ protein interactions enabling new insights regarding the macromolecular complexes involved in *P. vivax* protein–protein interactions.

## Methods

### Primer design

Primers containing a partial *attB* recombination sequence flanking a gene-specific sequence were used for amplifying genes of interest from *P. vivax* cDNA or gDNA (Table S1). For example, the forward primer 5′-**AAAGCAGGCT**TC*GAAGGAGATAGAACCATG*GAAACAGAAAGTTATAAGCAGC-3′, having the partial *attB* sequence (in bold) included a gene-specific portion (underlined) and Shine Dalgarno and Kozak consensus sequences to enable protein expression (italics); the reverse primer 5′-**AGAAAGCTGGGT**CTCCTGTTGTTCCAGGCTGTACC-3′ included a partial *attB* sequence and the gene-specific portion. The stop codon was removed to enable the PCR product to be fused in frame with a C-terminal GST tag. A set of universal primers containing complete *attB1* (GGGGACAAGTTTGTACAAAAAAGCAGGCTTCGAAGGAGAT) and *attB2* (GGGGACCACTTTGTACAAGAAAGCTGGGTCTCC) sequences were also synthesized. Regions overlapping the partial *attB* sequence from gene-specific primers are underlined.

To fuse *Pv*12 in frame with an N-terminal Halo tag, gene specific primers (underlined) were designed with the complete *attB* recombination sequence (in bold) (forward 5′-**GGGGACAAGTTTGTACAAAAAAGCAGGCT**CCACGTGCGATTTTAATG-3′; reverse 5′-**GGGGACCACTTTGTACAAGAAAGCTGGGT**C*CTA*GCCCTGCAGAACATTCGC-3′). The initiation (ATG) codon was deleted in each forward primer and the stop codon was maintained in all reverse primers (in italics).

The region encoding the ectodomain was selected for each gene, excluding signal peptide, transmembrane domain and glycosylphosphatidylinositol (GPI) anchor sequences (when present). For some proteins (such as *Pv*MSP1, *Pv*RBP1a, *Pv*DBP and *Pv*RON2), several fragments based on functional or previously studied regions were amplified (Table 1).

**Table 1:** *P. vivax* proteins selected for NAPPA array

Annotation	Pv gene ID	Time transcription^a^	Localization	Proteome data^c^	Fragments	Regions expressed
Target *P. vivax* proteins						
Merozoite surface protein 1 (*Pv*MSP1)	PVX_099980	TP7	Merozoite surface	X	2	^1350^D-F^1723^; ^761^G-E^1349^
Merozoite surface protein 4 (*Pv*MSP4)	PVX_003775	TP7			1	^30^I-S^248^
Merozoite surface protein 8 (*Pv*MSP8)	PVX_097625	TP2		X	1	^24^G-Y^462^
Merozoite surface protein 10 (*Pv*MSP10)	PVX_114145	TP7			1	^30^L-A^459^
6-Cysteine protein 41 (*Pv*41)	PVX_000995	TP7		X	1	^19^A-Q^383^
6-Cysteine protein 12 (*Pv*12)	PVX_113775	TP7		X	1	^27^T-G^336^
Asparagine rich protein (*Pv*ARP)	PVX_090210	TP7		X	1	^16^C-V^284^
Thrombospondin-related protein (*Pv*TRAMP)	PVX_123575	TP8	Apical/merozoite surface		1	^24^K-I^300^
Merozoite surface protein 5 (*Pv*MSP5)	PVX_003770	TP8	Micronemes/apical	X	1	^22^R-I^362^
Apical merozoite antigen 1 (*Pv*AMA1)	PVX_092275	TP8		X	2	^43^P-E^343^; ^43^P-^L487^
Duffy binding protein 1 (DBP1)	PVX_110810	↓ transcription^b^			3	^26^E-C^217^; ^198^T-D^524^; ^521^T-T^1000^
Reticulocyte-binding protein 1a (*Pv*RBP1a)	PVX_098585	TP8			4	^582^E-E^1457^; ^1549^F-G^1758^; ^1880^S-R^2229^; ^2245^S-E^2832^
Rhoptry neck protein 1 (*Pv*RON1)	PVX_000945	TP7	Rhoptry neck protein		1	^25^K-R^772^
Rhoptry neck protein 2 (*Pv*RON2)	PVX_117880	TP7		X	2	^735^G-L^1560^ and ^1554^L-V^2203^
Rhoptry neck protein 5 (*Pv*RON5)	PVX_089530	TP7		X	1	^23^F-W^500^ (*Pv*RON5A); ^50^N-P^1158^ (*Pv*RON5C)
Rhoptry neck protein 4 (*Pv*RON4)	PVX_091434	TP7			1	^25^F-I^756^
Rhoptry-associated protein 1 (*Pv*RAP1)	PVX_085930	TP7	Rhoptries		1	^2^T-Y^633^
Rhoptry-associated protein 2 (*Pv*RAP2)	PVX_097590	TP7		X	1	^22^H-H^382^
High molecular weight rhoptry protein 3 (*Pv*RhopH3)	PVX_098712	No data			1	^21^Q-F^599^
Rhoptry associated membrane antigen (*Pv*RAMA)	PVX_087885	TP7	Rhoptry body protein	X	1	^21^F-G^710^
Prey *P. vivax* protein						
6-Cysteine protein 12 (*Pv*12)	PVX_113775	TP7	Merozoite surface	X	1	^27^T-G^336^

^a^Transcription data from time points (TP) 1–9 of 3 *vivax* malaria isolates [82]

^b^Low transcriptional levels in all time points measured (TP1–TP9) [82]

^c^Data from proteomic analyses [51, 55]

### Cloning and subcloning

The first round of PCR involved 50 μL containing 25 μL 2× KAPA HiFi Ready Mix, 3.0 μL of each specific primer (5 μM concentration partial attB recombination sequence), 15 μL nuclease-free water and *P. vivax* cDNA or gDNA as template. PCR conditions for each gene involved an initial denaturing step at 95 °C for 3 min, followed by 35 cycles consisting of denaturing at 98 °C for 20 s, annealing at 58–60 °C for 30 s and an extension step at 72 °C for 1–2 min. A final extension cycle lasted 5 min at 72 °C. The product obtained from each gene was purified by Wizard SV Gel and PCR Clean-Up System (Promega), according to the manufacturer’s specifications. The second round of PCR involved 50 μL reaction including a set of universal primers (5 μM concentration), 25 μL 2× KAPA HiFi Ready Mix, 15 μL nuclease-free water and the purified product obtained in the first PCR to introduce the remainder of the *attB* sequence needed for recombination using the Gateway system (Invitrogen) [40]. The product obtained from each gene was purified and then used for BP recombination.

Obtaining the pDONR constructs involved transferring 100 fmol of each purified insert into the *attP* sequence-containing pDONR221 entry vector (150 ng/μL) in 2 μL BP clonase (Invitrogen). The reaction was incubated for 4 h at 25 °C and then used to transform One Shot TOP10 chemically competent *E. coli* (Invitrogen). The cells were plated onto LB agar-kanamycin and colonies were screened by PCR to identify positive clones. Five positive clones were sequenced with M13 forward and reverse primers to confirm the presence of the *P. vivax attL* gene-specific sequence.

LR reactions for transferring the pDONR221-containing *attL* sequence into *attR*-containing pANT7-cGST or PJFT7-nHalo involved 20 μL LR enzyme (Invitrogen) 1:1 ratio (donor:destination vector, each at 150 ng/μL) for 1–4 h at 25 °C. Transfer reactions were used to transform the DH5α competent *E. coli* strain (Invitrogen) which was then plated onto LB agar-ampicillin.

### Plasmid sequencing

The University of Salamanca’s (CSIC) Cancer Research Centre (Spain) sequenced all pANT7-cGST inserts in both strands using primers flanking the recombination regions (forward: 5′-TAATACGACTCACTATAG-3′; reverse: 5′-CCGCAAGCTTGTCATCAACCACTT-3′). Ten plasmids from the expression library were randomly selected to assess insert presence by restriction with BsrGI (New England) and to discard plasmid degradation.

The *pv12* gene cloned into pJFT7-nHalo was sequenced in both strands by using primers flanking the recombination regions (forward: 5′-CCCATTGTATGGGATCTGATC-3′; reverse: 5′-TGTTTCGCCATTTATCACCTTC-3′) at the sequencing service of the Cancer Research Centre (University of Salamanca-CSIC, Spain).

### Sample preparation, substrate functionalization and array printing

Sample preparation involved seeding 3 µg of each purified plasmid DNA in a 384-well microtitre plate and then incubating overnight at 40 °C to allow the DNA to dry; 12 µL master mix solution (containing 33.3 mg/mL bovine serum albumin (BSA), 2.5 mg/mL rabbit polyclonal anti-GST antibody and 2 mM Bis-(sulfosuccinimidyl) suberate (BS3)) was added to each precipitated plasmid DNA and then incubated for 30 min at 37 °C, following previously described protocols [35, 37]. Microscope glass substrates were functionalized with aminosilane, as previously described [41, 42].

The 384-well-plates and functionalized substrate slides were loaded into an Ultra Marathon robotic microarray spotter (Arrayjet Inc.) configured for simultaneously printing 48 samples (including the plasmid from each selected antigen and control samples), producing 150 µm features. cDNA-containing master mix samples (500 pl per sample containing approximately 3 ng plasmid DNA/spot) were printed by non-contact inkjet printer (UltraMarathon, Arrayjet). The printer was set up to print sub-arrays in 8 rows × 6 columns. Sixteen sub-arrays were printed per slide and each sub-array contained 29 cDNAs encoding *P. vivax* proteins and one cDNA encoding a *P. falciparum* protein. The printed arrays were then stored at room temperature in an airtight container with silica packets and protected from light. Several features were included in the array as negative controls, such as clean buffer, master mix without cDNA and master mix components independently (BSA or BS3 or anti-GST antibody).

### In situ protein expression using the NAPPA approach

Once the array had been constructed and cDNA evaluated, proteins were expressed using two CFPS protocols: a rabbit reticulocyte lysate (RRL) (Promega) or 1-step human coupled in vitro translation (HCIVT) (Thermo Scientific) systems. The IVTT lysate master mix was prepared with 200 μL reticulocyte lysate (Promega) containing 16 μL TNT buffer, 8 μL T7 polymerase, 4 μL -Met, 4 μL -Leu or -Cys, 8 μL RNaseOut (Invitrogen Inc.) and 160 μL DEPC water, following the manufacturer’s instructions. The master mix for HCIVT was prepared by mixing 88 μL HeLa lysate, 5.2 μL accessory proteins, 10.8 μL reaction mix, 1 μL leupeptin and 1 μL aprotinin (both at 1 µg/mL final concentration), as described by the manufacturer.

A HybriWell (Grace Biolabs Inc.) gasket was pressed onto the slides and each lysate master mix was individually added onto a slide through the gasket port. The HybriWell was gently massaged to spread the mix uniformly onto the array. Port seals were applied to both ports on the HybriWell to avoid evaporation. The arrays were incubated for 90 min at 30 °C and 30 min at 15 °C for protein expression and capture by the anti-GST polyclonal antibody. The HybriWell was then removed and the array washed three times with PBS for 5 min on a rocking platform.

### Analysing cDNA and protein display onto the arrays

Prior to functional assays, some control experiments were performed to ensure the quality of printing and protein expression on the arrays (QC assays).

Printed arrays were washed with phosphate-buffered saline (PBS), pH 7.4, for 15 min with gentle shaking, followed by a brief washing step with deionised water for 1 min. The array surface was blocked with 20 mL Superblock PBS for 1 h at room temperature (RT) with gentle shaking followed by a 5 min wash with deionised water. The arrays were dried under a stream of filtered compressed air [40]. Blocked slides were incubated with 150 μL/slide of 1:600 (v/v) diluted PicoGreen dye (Invitrogen Inc.) for staining the cDNA to evaluate DNA printing quality. The slides were scanned using a ProScanArray HT scanner (Perkin-Elmer) and the resulting images were analysed and quantified using GenePix Software version 6.0 (GenePix).

The slides were then incubated with Superblock PBS (Pierce) for 1 h at RT to ascertain whether protein expression had been universal throughout the whole array and then incubated with mouse anti-GST antibody (Cell Signaling Technologies Inc.) in Superblock PBS at 1:200 (v/v) dilution for 1 h at RT to evaluate protein expression throughout the whole array. After three 5-min washes with washing buffer (PBS 5% milk + 0.2% tween 20), the slides were incubated with HRP-linked anti-mouse IgG (Amersham) for 1 h at 1:200 dilution and washed again three times with PBS (5 min/wash). Signal was developed by incubating with 200 μL/slide tyramide signal amplification reagent (Perkin-Elmer) for 10 min at RT. The slides were then rinsed with deionised water, dried using compressed air and scanned (array images were analysed as described above).

Query cDNA was co-expressed by supplementing the IVTT master mix (for RRL) with 1 µg of cDNA encoding the *Pv*12 protein with a Halo tag to check in situ expressed *P. vivax* protein functionality (Table 1 and Additional file 3: Table S1); the interaction was then indirectly detected by incubation with anti-Halo antibody (Promega).

*Pv*12 interaction with the remaining proteins on the array was assessed by triplicate. Taking into account that each array contains 16 sub-arrays, each recombinant protein (all the 29 of them) is represented 16 times in each array.

### Processing microarray data and statistical analysis

Computational processing of arrays began by acquiring the images, followed by analysing each spot [21]. Data was normalized (taking sub-array as analysis unit) by dividing the mean for the master mix (corrected for sub-array) by the difference in value of the spot’s total fluorescence minus the average value for empty spot and/or master mix. If such value was > 1 this meant the presence of DNA or protein in the spot. The mean of the previously normalized values was also obtained for analysing each clone’s expression; if this was > 1 it indicated protein presence [21, 35, 37].

## Results

### Designing a *Plasmodium vivax* NAPPA

**Fig. 1:**
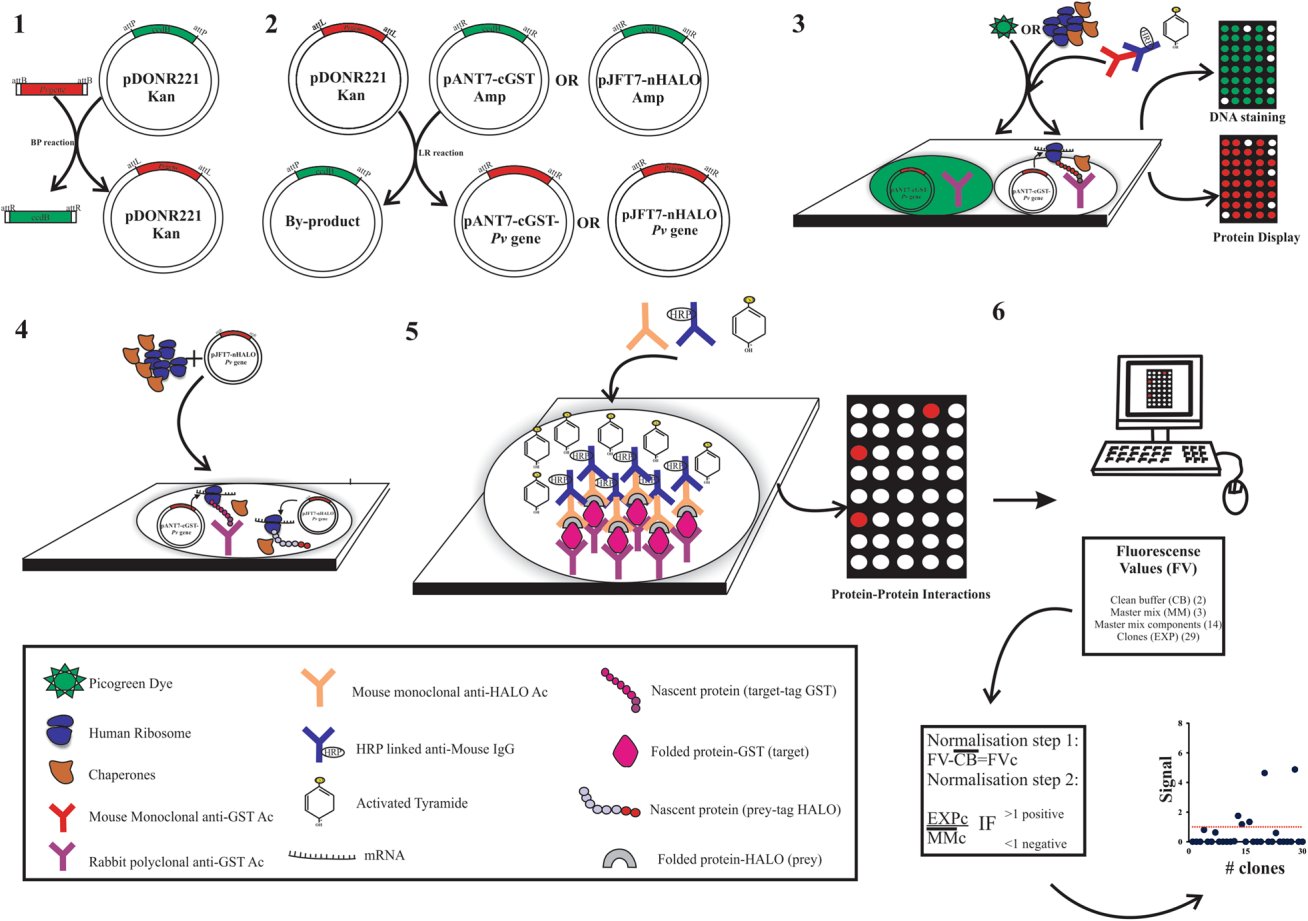
Outline of NAPPA protocol for detecting PPI in *P. vivax*. Step 1. Cloning: 35 DNA fragments from 20 *P. vivax* genes were amplified, adding attB recombination sequences for each one. Entry vector (pDONR221) recombination involved using BP clonase. Step 2. Sub-cloning: a second recombination involved positive clones (pDONR-Pv gene) and destination vectors (pANT7-cGST or pJFT7-nHalo) using LR clonase. Step 3. Printing and quality control: *P. vivax* plasmids were mixed with BSA, a crosslinker and rabbit polyclonal anti-GST antibody, and printed in spots on aminosilane-coated glass slides. After blocking, PicoGreen dye or IVTT (RRL or HCIVT) were used for staining slides. PicoGreen readings were used to calculate intra- and inter-slide correlation for assessing DNA printing quality. IVTT involved detecting captured proteins using mouse monoclonal anti-GST (primary) and HRP-linked anti-mouse IgG (secondary) antibodies, using tyramide as activated substrate. The amount of positive and negative spots was used to estimate expression performance. Step 4. Prey/bait co-expression: The IVTT system (RLL) was added together with prey plasmid to the array containing bait plasmid. Step 5. PPI detection: The PPI were revealed using mouse anti-Halo monoclonal (primary) and HRP-linked anti-mouse IgG (secondary) antibodies, using tyramide as activated substrate. Step 6. Data analysis: Fluorescence values were normalized to estimate positive signal values (greater than 1) for DNA printing and protein expression

Twenty genes encoding potential *P. vivax* vaccine candidates were selected based on several criteria (Table 1), such as the shared homology between *P. vivax* and *P. falciparum* sequences, functional evidence regarding target cell binding, antigenicity and/or immunogenicity, transcription during the last hours of the intraerythrocytic cycle (TP7–TP9) and evidence of expression in late schizonts and localization on parasite surface or apical organelles. Thirty-five PCR products were obtained from 20 selected *P. vivax* gene fragments, 29 of which were successfully cloned using Gateway technology (83% efficiency) (Table 1 and Additional file 1: Fig. S1). This methodology used pDONR221 and two destination vectors (pANT7-cGST or PJFT7-nHalo) to transfer encoding sequences from the first plasmid to the second one (Fig. 1). pANT7-cGST contained a GST protein enabling nascent protein capture by a fixed polyclonal antibody against GST on the array, whilst query protein was cloned in pJFT7-nHalo (N-terminus Halo tag) and detected by monoclonal anti-Halo tag antibodies (Fig. 1).

**Fig. 2:**
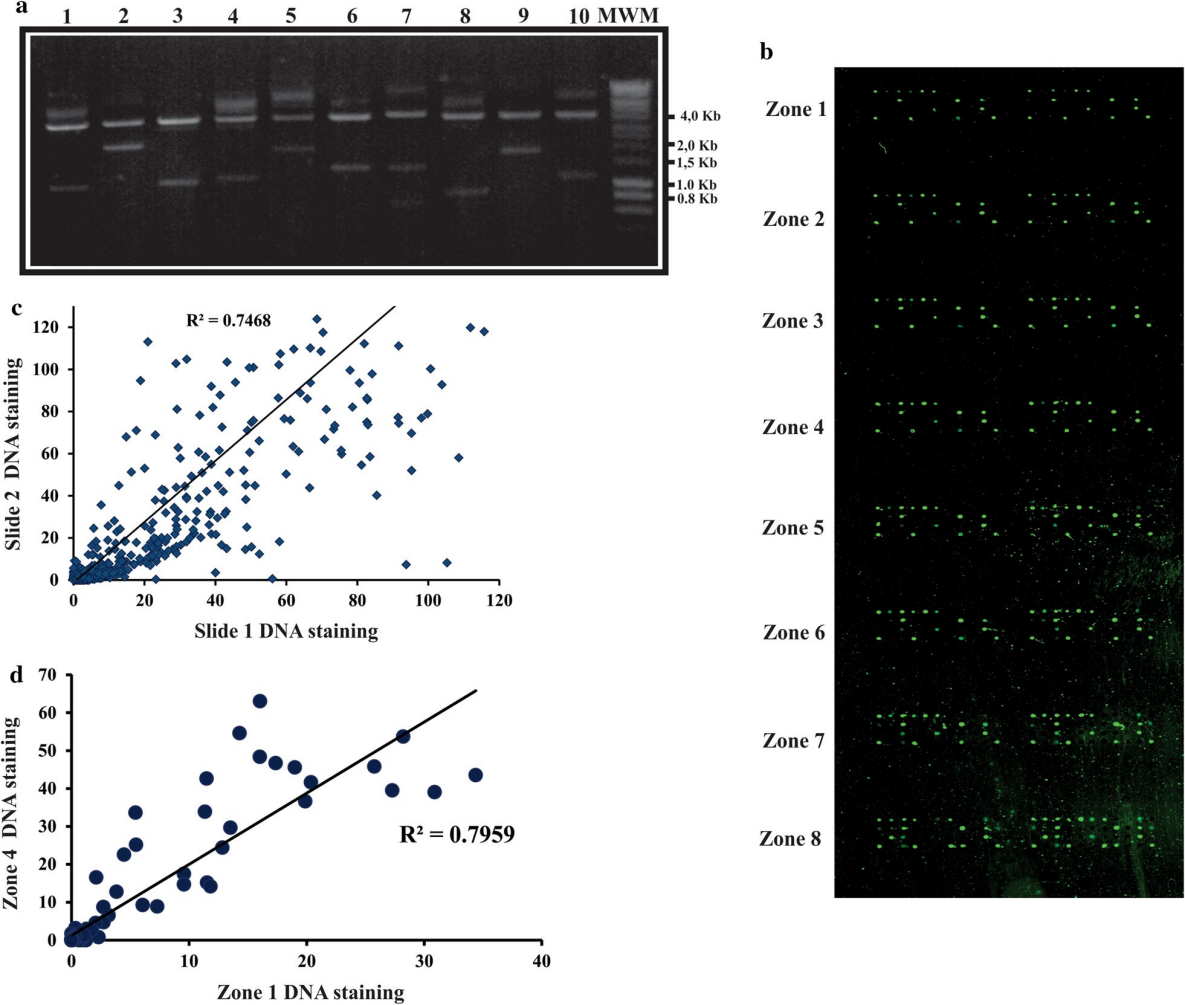
Analysing printed DNA reproducibility. **a** Analysis of expression library quality by enzymatic digestion. The BsrGI enzyme was used for digestion; two DNA fragments were produced. The 4 kbp band corresponds to the linearized vector, whilst the smaller fragment corresponds to the released insert. The presence of more than one band below 4 kbp indicate that the insert contains restriction sites for BsrGI. MWM: molecular weight marker. At least two fragments were observed in all cases. **b** Scanning images showing the spots (i.e. DNA printed onto cell surface before expression). The Figure shows 16 sub-arrays (containing 29 clones and negative controls) for each array. Each region is represented by two sub-arrays. **c** Inter-slide reproducibility showing the relationship between normalized PicoGreen signals for clones printed in different arrays. **d** Intra-slide reproducibility showing the relationship between clones printed on different sub-arrays

This experimental approach has more advantages than traditional cloning methods because it does not require the use of restriction enzymes and site-specific recombination enables DNA fragments to be correctly cloned in frame with GST or Halo tags, thereby simplifying cloning [43, 44]. All the clones from this expression library were full-sequence validated and their sequences were analysed by BLAST to confirm their identity. A set of 10 clones (randomly selected from the expression library) were digested by BsrGI to verify insert presence and ascertain plasmid DNA quality (Fig. 2a). Expression library quality was validated and the clones were then ready for designing the NAPPA array.

Glycerol concentration and drop number were tested (data not shown) to homogenize and avoid aberrant effects as master mix component complexity (BSA, cDNA, cross-linkers, anti-tag antibody) could affect non-contact deposition and because viscosity plays a critical role. Optimal conditions were found to be 50% (v/v) glycerol and 5 drops per spot. The cDNA staining signal in these arrays was > 1 in all the spots after normalization against control spots (master mix components without cDNA). This meant that all cDNAs in the expression library had been successfully deposited on the array by the non-contact strategy. For example, cDNA staining signal Duffy Binding Protein region-1 (DBP-RI) was detected in 94% of the spots.

The normalized signal obtained from printed cDNA was then evaluated across intra- and inter-arrays to evaluate robustness and reproducibility (Fig. 2), good intra-array (R^2^ = 0.80) and inter-array reproducibility (R^2^ = 0.75) being observed (Fig. 2). These results should guarantee consistent reproducibility levels for further studies with this *P. vivax* expression library.

### Self-assembling protein array displaying *Plasmodium vivax* proteins

**Fig. 3:**
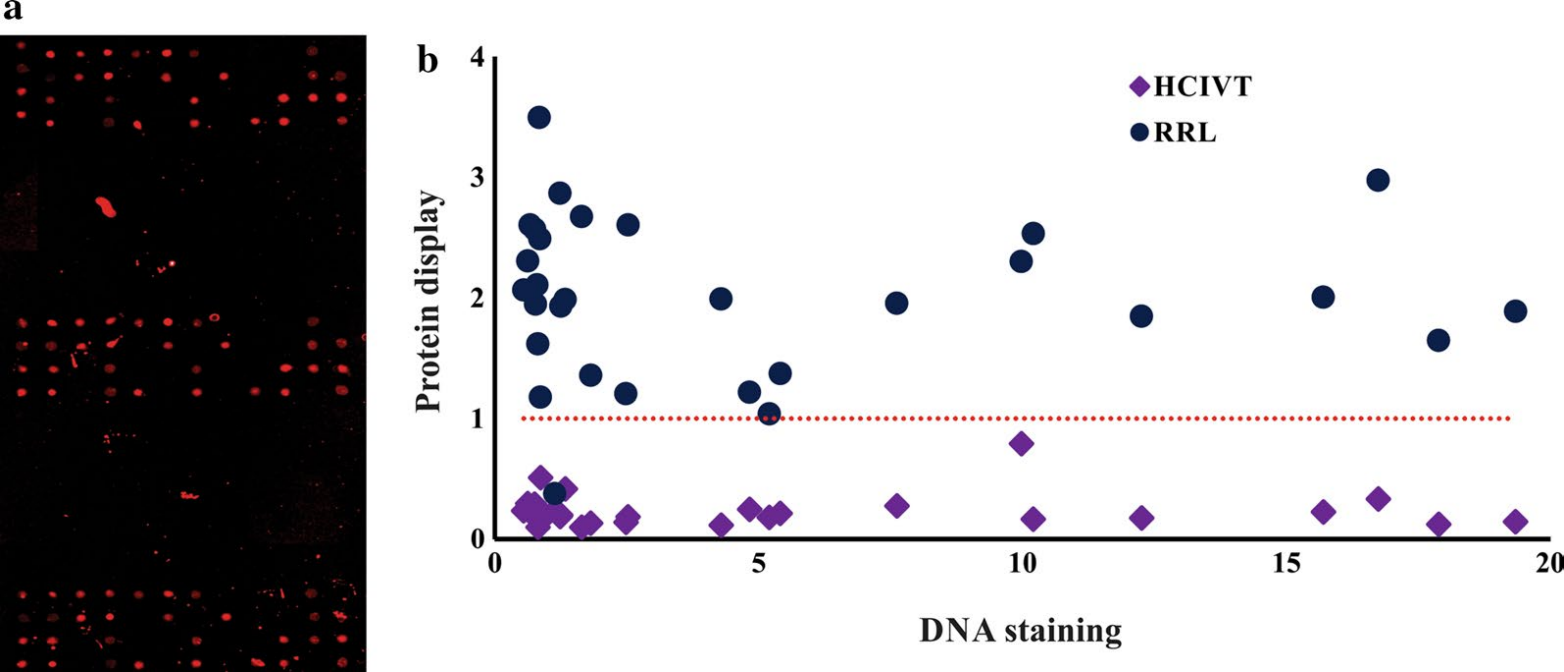
Analysing *Plasmodium vivax* protein expression using RRL or HCIVT in NAPPA arrays and Western blot. **a** Scanning images showing the spots for three sub-arrays for proteins expressed with the RRL system after incubation with anti-GST. **b** Comparing *P. vivax* antigen expression with rabbit reticulocyte lysate (RRL) and 1-step human coupled IVT (HCIVT). The relationship between normalized cDNA signal and normalized protein signal for each expression system is shown. Values greater than 1 were considered positive

Spotted *P. vivax* cDNA was expressed in situ with RRL and protein presence was detected by anti-GST monoclonal antibody (as described in “Methods”). This led to signals being detected for all cDNAs in the *P. vivax* expression library in all printed cDNA (100% efficiency) (Figs. 1, 3a, b, Additional file 2: Fig. S2). A second HeLa lysate-based in vitro expression system (HCIVT) was used with the same NAPPA *P. vivax* array. Nascent protein (C-terminal GST tag) was also detected by anti-GST monoclonal antibodies (as described in “Methods”). Figure 3b depicts the HCIVT system normalized signal; no expression was detected using this system although a signal was detected in 10% of the spots for the following 17 proteins (normalized signal > 1): (*Pf*MSP1_83_, *Pv*41, *Pv*MSP10, *Pv*MSP1_42_, *Pv*MSP4, *Pv*MSP5, *Pv*MSP8, *Pv*RAP2, *Pv*RBP1a-RIV, *Pv*RBP1a-RI, *Pv*RBP1a-RII, *Pv*RBP1a-RIII, *Pv*RON1, *Pv*RON2-RII, *Pv*RON2-RIII, *Pv*RON4 and *Pv*RON5C).

### *Plasmodium vivax* in situ protein–protein interaction studies

Recent protein–protein interaction screening has involved using Avidity-based extracellular interaction screening (AVEXIS) technology to characterize interaction between 34 *P. vivax* Mrz proteins (bait and preys) [45]. This intra-library AVEXIS was only able to identify three *P. vivax* protein–protein interactions in bait-prey orientation, one such being *Pv*12 interaction with *Pv*41 protein. *Pv*12 protein interaction was then tested with *P. vivax* proteins displayed on the array to evaluate whether NAPPA was working properly; *Pv*12 is a GPI-anchored 6-Cys protein expressed on parasite schizont surface and associated with DRM complexes [46], this being where the formation of several of the parasite’s protein complexes involved in host cell interactions take place [47].

**Fig. 4:**
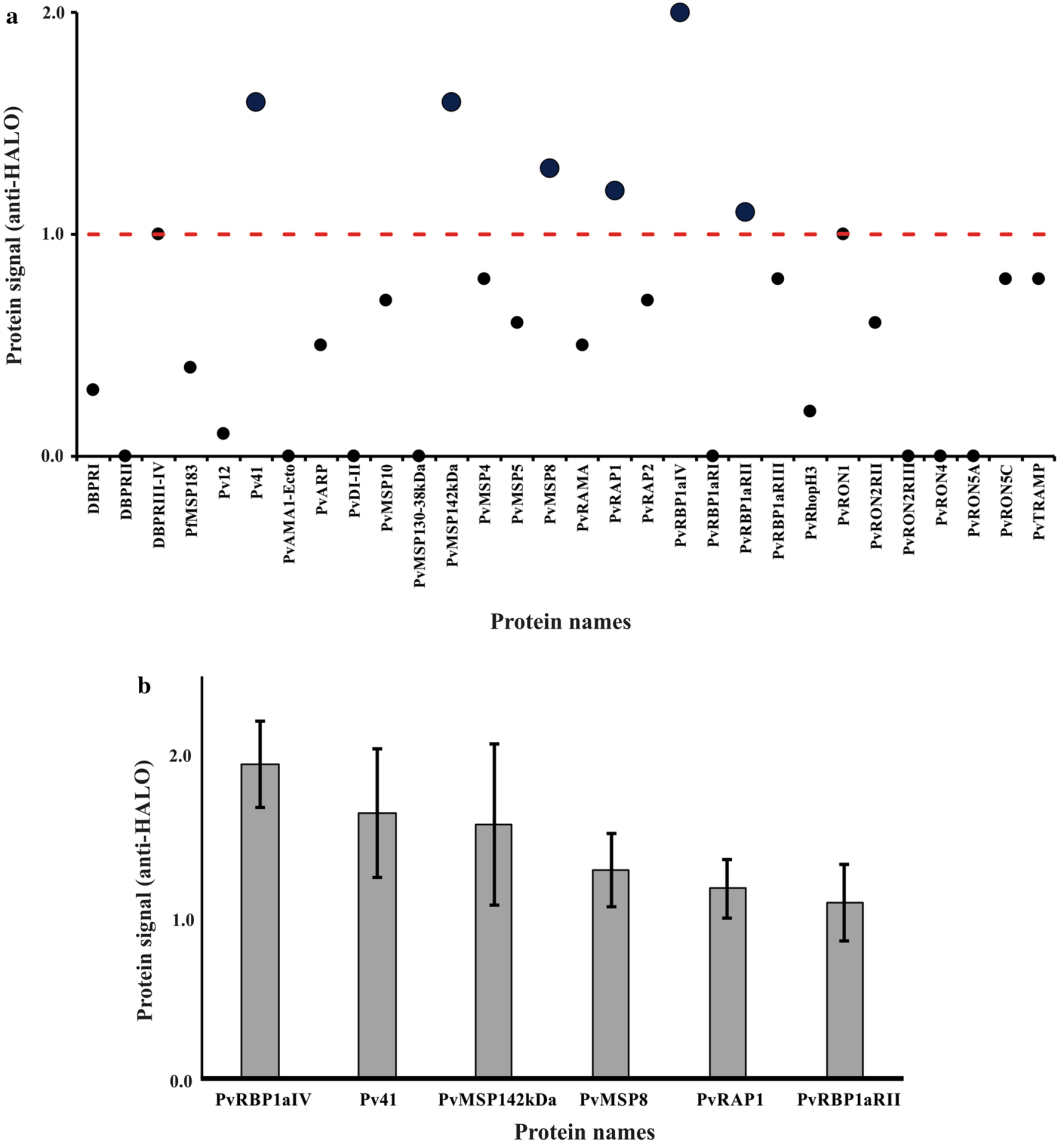
NAPPA functional assessment of *Plasmodium vivax* proteins. **a** The *Pv*12 protein was used as query for evaluating its interaction with 29 *P. vivax* protein fragments displayed on the array and one *P. falciparum* protein (*Pf*MSP1_83kDa_). Query DNA encoding an N-terminal Halo tag was added to reticulocyte lysate for co-expression with bait proteins. The interaction signal was determined with anti-Halo antibodies. Normalized values are shown for 30 clones evaluated on the array. A median value > 1 was considered positive interaction. **b** Median value ± standard deviation of proteins interacting with *Pv*12

*Pv*12 was then cloned and sub-cloned (pDONR221 and pJFT7-nHalo) as described in “Methods” (Table 1 and Additional file 3: Table S1). *Pv*12 as query protein was co-IVTT expressed with a *P. vivax* cDNA library array for in situ protein interaction studies; interactions were detected by anti-Halo tag antibody (Fig. 1), showing that *Pv*12 interacted with five *P. vivax* proteins located on surface membrane and in rhoptry organelles, mainly with *Pv*RBP1a region IV and *Pv*41 proteins (Fig. 4).

## Discussion

Most pathogen (i.e. *Plasmodium*) invasion is PPI-mediated, leading to stable or transient molecular complex formation [48]. Identifying and characterizing these types of interaction strongly suggests a functional relationship between participating proteins and enables understanding the biological mechanisms (replication, transcription, metabolism and invasion) used by microorganisms for invading and infecting target cells. Blocking PPI has been suggested as a target for action against pathogens by designing new drugs or small binding molecules targeting cell surface or the contact region between proteins [49, 50].

NAPPA is one of the chosen methodologies for characterizing PPIs. The first step in this study was thus the rational selection of content and query and evaluating whether a variety of such *P. vivax* proteins could be displayed by this format (Fig. 1). Twenty proteins were selected based on criteria described in the Results section (Table 1) [15, 51–55] and functional evidence of these antigens in *P. vivax* and other species (i.e., *P. falciparum*) during Mrz invasion of target cells [15].

Selected antigens (Table 1) were subdivided into three groups according to their subcellular location on Mrz surface proteins and proteins located in the micronemes and in the rhoptries. Mrz surface proteins are primarily exposed on Mrz plasma membrane during invasion and some of them are involved in weak, low-affinity interactions with receptors on RBC membrane for selecting a specific target cell [3, 56]. Four proteins were selected from the Mrz surface (MSP) group (*Pv*MSP-1, -4, -8 and -10); all of them are membrane-anchored via glycosylphosphatidylinositol (GPI) and expressed on *P. vivax* schizonts [57–59]. MSP1 is the major MSP and has been implicated in many *P. falciparum* receptor–ligand (glycophorin A, B and 3 and heparin) and protein–protein interactions (MSP6-MSP7-RhopH3-RAPs) [7, 8, 11]. It has been the object of significant antigenic and immunological studies highlighting its importance as *P. falciparum* and *P. vivax* vaccine antigen candidate [60, 61]. The other surface antigen group selected here included the *Plasmodium*-specific 6-Cys family containing a cysteine-rich domain, the 6-cysteine or s48/45 domain [62]. This *Plasmodium*-specific family’s proteins are expressed in a stage-specific manner and perform important functions during life-cycle (gamete, Spz or Mrz) stages. P12 and P41 proteins have been characterized as blood-stage 6-Cys proteins in *P. falciparum* and *P. vivax* and have been seen to form a stable complex on the infective DRM-associated Mrz surface [45, 63].

Microneme and RON proteins are involved in high-affinity interactions initiating parasite entry to RBC. Native *Pv*RBP1a is colocalized on Mrz microneme with *Pv*DBP [64]; whilst RBP proteins are responsible for the specificity of *P. vivax* Mrz binding to reticulocytes, *Pv*DBP is involved in *P. vivax* selectivity for invading Duffy antigen cells expressed on reticulocyte surface [65]; both proteins have been considered important vaccine candidates [60]. The AMA-1 protein has been implicated in macro-complex formation, together with RON2, -4 and -5 proteins for establishing the TJ necessary for parasite mobilization inside its target cell [66]. The content of the rhoptries involved in PV formation is then discharged onto a host/target cell for successful entry [67]. Many proteins have been seen to be involved in this last step during Mrz invasion, such as RhopH3, RAP1 and RAP2. *P. falciparum* RAP1, RAP2 and RAP3 form a low molecular weight complex in the bulb of the rhoptries, this being invasion-inhibitory monoclonal antibodies’ target in vitro [68]. Other proteins such as *Pv*TRAMP, *Pv*RON1 and *Pv*ARP have been recently identified and characterized in *P. vivax* schizonts and have been recognized by *P. vivax*-infected individuals’ serum [69–71].

The array was designed to display cDNA encoding *P. vivax* proteins several times so as to have several replicates but mostly to avoid cross-contamination across features and highly homogeneous spot morphology. A non-contact printer was used to create these arrays which, apart from the requirements described above, had an advantage regarding the amount of cDNA per master mix (3 µg) compared to 10-15 µg cDNA/master mix previously reported in arrays constructed by microcontact printers [37].

One of the greatest challenges when working with protein microarray formats is being able to guarantee the proper folding and posttranslational modifications. The proteins produced and purified in heterologous systems, especially *E. coli*, may either lack modifications or display unnatural ones. The CFES, particularly the eukaryotic ones, offer an open, customizable and versatile system that contains the machinery for introducing the desired modifications such as acetylations, glycosylations, phosphorylations and signal peptide processing, amongst others [72–74]. It is worth highlighting that eukaryotic extracts also contain mammalian ribosomal machinery and the presence of chaperones, like hsp90, hsc70 and others, which may encourage folding [27]. Different studies in *Plasmodium* have shown that eukaryotic expression in live cells (i.e. HEK293 cells) or CFES (i.e. WGE and HeLa cells) are able to produce properly folded recombinant proteins. As an example, 39 *P. vivax* antigens have been expressed in HEK293 cells, most of which were recognized by pooled plasma from 14 patients having acute vivax malaria, the seroreactivity decreased at least 20% when the recombinant proteins were heat-treated, indicating that they contained conformation-sensitive epitopes [45]. Another study has shown that *P. vivax* proteins from both merozoite and sporozoite forms expressed in WGE are better recognized by sera of *P. vivax* patients than the same proteins when produced in *E. coli* [30]. Moreover, when a *P. vivax* metalloprotease, encoded by the PvHSP28 gene, was expressed with a RRL system, it retains all its enzymatic activity [75]. The RRL system is one of the most used NAPPA expression systems, producing hundreds of proteins including various microorganisms’ proteomes and more than 1000 human proteins [29, 36, 76]. The present study has shown the feasibility of producing *P. vivax* antigens from surface membrane, rhoptries and micronemes in array format with the RRL system (Fig. 3). Although previous studies in *Plasmodium* have shown that HeLa cell lysates were able to express four *P. falciparum* antigens with a varying degree of solubility, when the HCIVT system (based on HeLa cell lysates) was here used in array format, the expression of the 29 *P. vivax* fragments was lower than that obtained using the RRL system (Fig. 3). However, further experiments are required to optimize HCIVT-expressed array performance regarding *P. vivax*; this could include increasing lysate concentration, expression time, temperature or adding protease inhibitors.

When *P. vivax* protein–protein interactions were measured it was found that *Pv*12 protein used as bait interacted with *Pv*41, as previously described using AVEXIS technology [45]. AVEXIS is based on measuring protein–protein interactions between bait (biotinylated and captured by streptavidin-coated wells) and prey proteins (enzymatically tagged and containing a pentamerization domain to increase interaction avidity) [77]. This technology has been used to ascertain Rh5 interaction with the Basigin receptor [78], Rh5 interaction with *Pf*113 protein [79] and recently for evaluating *P. vivax* protein–protein interactions [45]. These studies were only able to detect three interactions between 34 bait and prey proteins (i.e. very few interactions compared to those identified in *P. falciparum*) [45, 80]. Although AVEXYS can increase avidity, prey pentamerization can cause steric hindrance affecting real detection of protein–protein interactions.

NAPPA detected interaction between *Pv*12 and other MSP (in addition to *Pv*12–*Pv*41 interaction), such as *Pv*MSP1_42kDa_ and *Pv*MSP8 and proteins located in the apical organelles, such as *Pv*RAP1 and *Pv*RBP1a (Fig. 4), which confirms the ability of this technique for detecting binary interactions in *P. vivax.* Although six interactions with *Pv*12 were detected, this does not necessarily mean that all of them have an effective chance of occurring, due to time or spatial differences in the expression of such proteins so, methodologically, such interactions must be confirmed by using other techniques such as immunoprecipitation, pull down assays or isothermal titration calorimetry. On the other hand, it is not that strange detecting interactions amongst proteins coming from different sub-cellular localization in *Plasmodium*, as an example, it has been reported that the *P. falciparum* apical merozoite antigen-1 (*Pf*AMA-1), originally localized in the micronemes, is able to interact with proteins expressed in rhoptry necks, leading to the tight junction formation that the parasite establishes with the target cell [66]. Moreover, it has been described that the *P. falciparum* P113 protein, anchored to the Surface membrane, specifically interacts with Rh5, expressed in the rhoptries [79]. Previous studies have described *Pv*12 forming part of DRMs [46], a platform where many Mrz invasion-associated proteins are organized into multi-protein complexes [47], thereby suggesting that *Pv*12 could be involved in forming a complex between parasite proteins. It is worth stressing that, in addition to NAPPA’s advantages regarding expression, in situ co-expression of analyte and query and RT storage, it has higher sensitivity than other protein–protein interaction measurement techniques, mainly because the protein–protein complex is only found in the small microspot area, resulting in increased high local signal. Although few molecules can be captured in the microspot, high density molecules can be obtained in it [81].

## Conclusions

NAPPA is a high-performance technique for evaluating interactions in *P. vivax*, offering a new and useful alternative for studying the biology of this difficult-to-culture parasite. It enables bait and query co-expression in situ, thereby enabling interactions to be analysed rapidly and reproducibly. NAPPA seems to be a flexible approach for identifying key PPI in full- or targeted-proteomes. This article has highlighted the NAPPA and RRL-based expression system for the successful and reproducible expression of *P. vivax* proteins which might be involved in *P. vivax* Mrz protein–protein interactions. The query protein (*Pv*12) was able to interact with parasite surface membrane- and apical organelle-derived Mrz proteins. This technique will facilitate studying therapeutic targets and clarifying protein–protein interaction mechanisms.

## Additional files

**Additional file 1: Fig. S1.** A representative sample of *P. vivax* gene amplicons.

**Additional file 2: Fig. S2.** Protein subarray map. The localization of each construct and control in each sub-array is shown. Each sub-array contains 48 spots in total and each array contains 16 sub-arrays. Controls include printing with just clean buffer, BS3, BSA+BS3, cDNA without master mix, polyclonal anti-Halo antibody, anti-GST monoclonal antibody or anti-Halo monoclonal antibody. A sub-array of protein expression using the RRL system is shown below. Anti-Halo antibodies were printed to capture the Pv12 protein on the array.

**Additional file 3: Table S1.** List of primers used, amplicon size, expressed products’ molecular weight and destination vectors for the genes analysed.
