# Supplementary figures and images for: Self-assembling functional programmable protein array for studying protein–protein interactions in malaria parasites
Source: Malar J. 2018 Jul 17;17:270. doi: 10.1186/s12936-018-2414-2 (PMC6050706; doi:10.1186/s12936-018-2414-2)

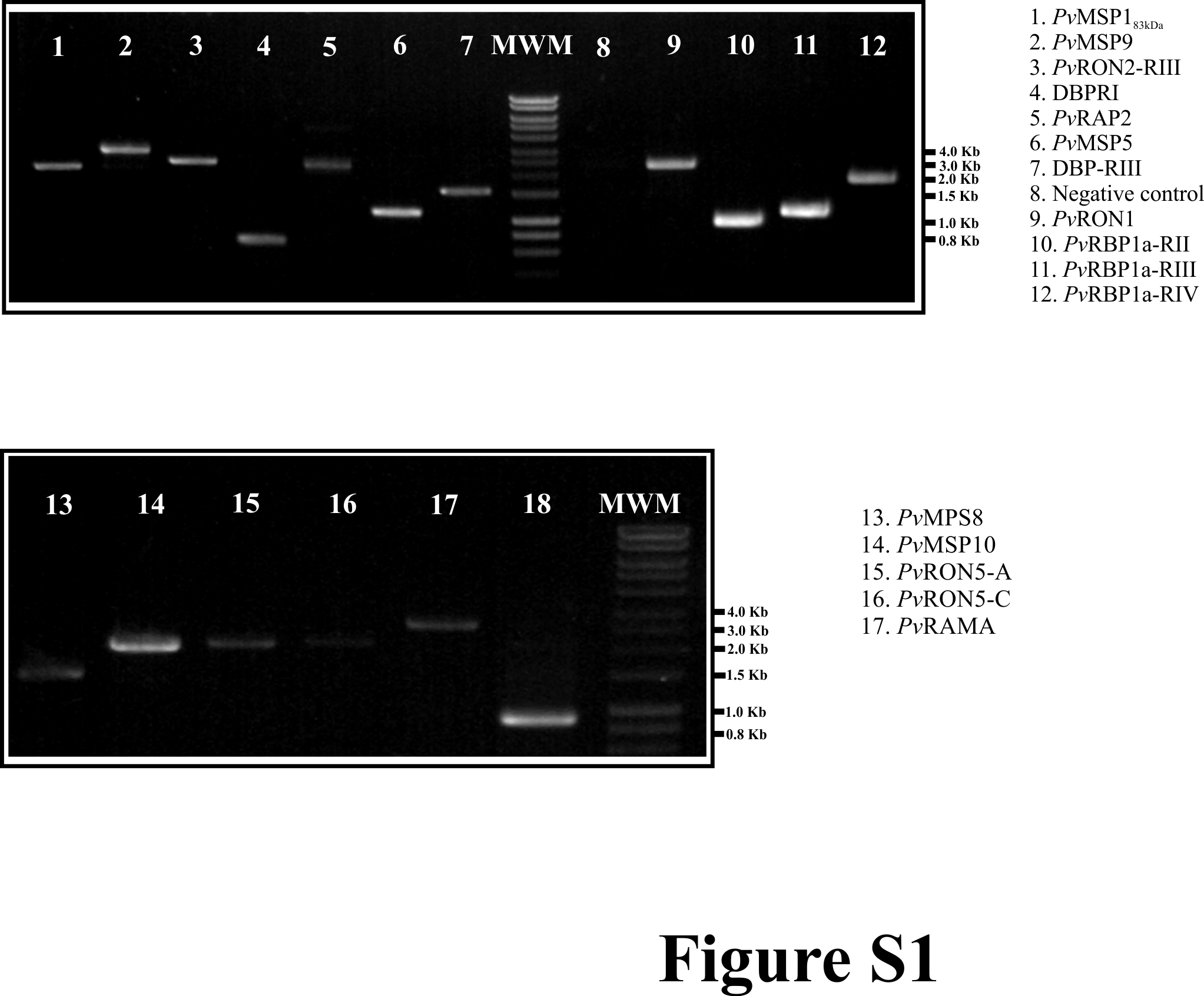

Supplement: Supplementary file 1 — Additional file 1: Fig. S1. A representative sample of P. vivax gene amplicons. [file 12936_2018_2414_MOESM1_ESM.tif]

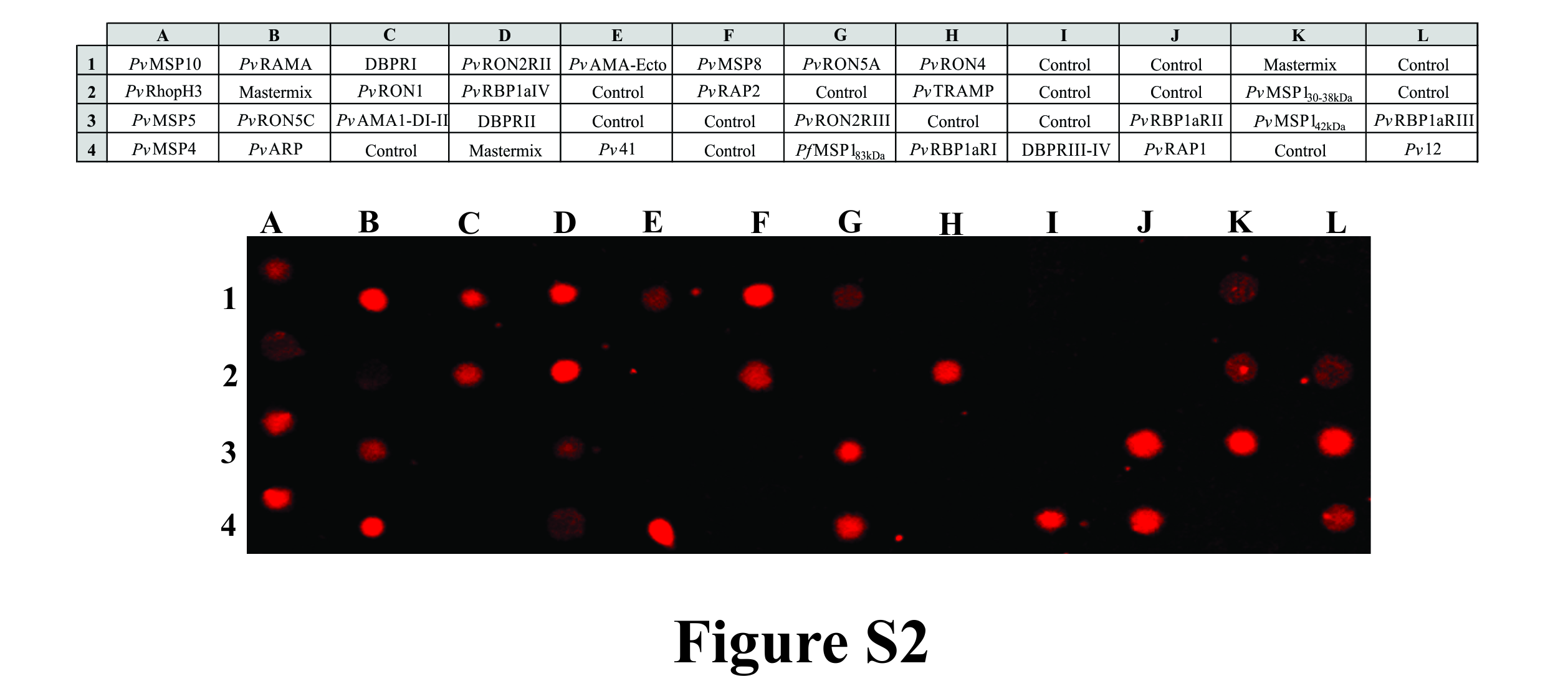

Supplement: Supplementary file 2 — Additional file 2: Fig. S2. Protein subarray map. The localization of each construct and control in each sub-array is shown. Each sub-array contains 48 spots in total and each array contains 16 sub-arrays. Controls include printing with just clean buffer, BS3, BSA+BS3, cDNA without master mix, polyclonal anti-Halo antibody, anti-GST monoclonal antibody or anti-Halo monoclonal antibody. A sub-array of protein expression using the RRL system is shown below. Anti-Halo antibodies were printed to capture the Pv12 protein on the array. [file 12936_2018_2414_MOESM2_ESM.tif]
